# Promote older adults active travel on neighborhood streets: an observation study in Daokou ancient town in China

**DOI:** 10.1186/s12877-025-06321-w

**Published:** 2025-10-21

**Authors:** Zhe Wang, Hongyi Lv, Guoxiang Li

**Affiliations:** 1 Department of Architecture, Henan University, Zhengzhou, China; 2 Department of Architecture, Zhengzhou University, Zhengzhou, China

**Keywords:** Active living, Physical activity, Design for aging, Social engagement, Person-environment.

## Abstract

**Background and objectives:**

Seeking to extend previous research on older adults’ active travel, this paper focused on the travel duration and neighborhood streets. With an emphasis on the older adults and streets in ancient towns, empirical data were collected in Daokou Ancient Town in China. The aim is to identify environmental attributes promoting active travel.

**Research design and methods:**

Non-participant observation was conducted for six weekdays on three neighborhood streets. Twenty street factors were measured and classified into four categories in terms of active travel promotion. Focusing on older pedestrians, 871 cases of active travel were recorded. Two-level ANOVA tests were conducted to identify the differences in active travel duration by street, with significant personal or social variables in control.

**Results:**

The duration of older adults’ active travel significantly varied by neighborhood street. Those who engaged in social interaction during active travel had longer travel durations. Among the healthy pedestrians in their 60s, for both females and males, irrespective of their social engagement, active travel durations were significantly longer on the streets with more age-and social-friendly features (p < = 0.01). The differences were no longer statistically significant in those aged 70 + or with health issues, whereas they spent more time staying on the streets.

**Discussion and implications:**

Age- and social-friendly neighborhood streets contributed to longer durations of active travel in older adults. Social engagement played an important role in their active travel. Street affordances for both the physical and social attributes of active travel should be created. More research is needed for those aged 70 + or with health issues.

## Background

Population aging is a global phenomenon and active living, especially active travel, has been widely suggested to older adults for health and quality of life. The World Health Organization recommends older adults engage in moderate-intensity physical activity such as walking for a minimum of 150 min per week [1]. The amount of time that older adults spend walking in their neighborhoods contributes to their independence and reduces the costs of cumulative long-term care [2, 3]. However, associated with age-related limitations, older adults are less likely to reach the recommended level of active travel. It has been found that the hours older adults spend sedentary ranges from 5.3 to 9.4 per waking day [4]. How to encourage and support older adults to engage in active travel needs attention. As an essential measure of active travel, the duration should be of high interest to older adults, designers, planners, and policymakers if promoting active living. It needs to be investigated with attention to the process of their active travel and the places where they travel.

### Process of active travel in older adults

The research phase ‘active travel’ originated in the literature, with an emphasis on the healthy outcomes of active living. From the perspective of transport, a recent definition of active travel is the ‘travel in which the sustained physical exertion of the traveler directly contributes to their motion’ [5]. The former focuses on health outcomes and the latter focuses on the modes of physical exertion for motion. Both have overlooked the process of active travel, which is considered important to older adults and involves a group of behaviors with both physical and social attributes.

Active travel among older adults has its special characteristics related to the travelers’ advanced ages. Influenced by age-related functional declines, older adults’ active travel involves low- or moderate-intensity physical activities, such as walking or cycling at arelatively low speed [6, 7]. Facilitated by the low-speed modes, their active travel typically offers time to interact with the surrounding environments and people. Since many older adults have unmet information needs associated with their generally reduced social contacts outside home, the time and opportunities to interact with the outside are of high importance to them [8].

Based on surveys and observations of older adults in both ancient towns and urban cities, researchers found that their active travel included not only physical activities for motion but also social activities [9, 10]. The modes of their active travel are hybrid, blurring lines between transport, recreation, leisure, and exercise mobilities [5]. For instance, older adults like to chat with people they run into while walking. Seeing others of a similar age group engaging in physical activity encouraged them to participate in physical activity [11]. These activities are integrated with each other. Social engagement during active travel, via either visual communication or dialogue, provides opportunities for older adults to access the world outside their home and reduces the feeling of loneliness, leading to promoted self-efficiency and mental well-being [12, 13]. Thus, the definition of active travel in older adults can be refined as the travel in which the sustained physical exertion of the older traveler directly contributes to their motion, typically at a low speed and frequently integrated with social activities.

Active travel can also serve utilitarian purposes, seamlessly integrating physical activity into daily tasks such as walking to a grocery store or commuting to work. This dual-purpose approach not only enhances health by promoting physical activity but also decreases reliance on traditional oil-based transportation, which poses risks to environmental sustainability and public health. By substituting vehicle-based travel with active modes of transport, such as walking or cycling, individuals can simultaneously improve personal well-being and support environmental conservation.

The engagement of regular active travel is facilitated by the generally flexible schedules after retirement. Many older adults’ activities in a day are no longer bounded to work but center on daily living. The schedule of having breakfast, lunch, and dinner has an important position in their daily living [14]. It may influence the time of their active travel, which can be viewed as a social factor involved in active travel. To investigate active travel in older adults, these social considerations need to be included in research.

### Places where older adults engage in active travel

According to the widely accepted environment-behavior theory and the extensive empirical findings in this field, one of the most important determinants of how long people travel is where they travel [15]. As age increases, the geographic radius of older adults’ daily-living activities generally diminishes. Many older adults spend the majority of their day at home. The importance of external residential environment to older adults’ health and wellbeing has been highlighted by researchers, including environmental navigation, mobility, and traffic [16]. The proximate neighborhood environments are the most convenient places for them to engage in active travel. The most popular type of active travel among older adults is walking and they are more likely to be healthy and engage in social activities in age-friendly and greenery neighborhoods [9, 17, 18].

Neighborhood streets provide walkways to pedestrians and can promote or hinder their active travel [19]. Some neighborhood attributes promote walking, such as sidewalks in good condition, routes and destinations for walking, seating areas, and safety from traffic and crime [20, 21, 22, 23]. On the other hand, crime, littering, vandalism, and decay are barriers older adults have to face as being a pedestrian in public spaces and negatively correlated with their walking [24, 25]. Focusing on older adults, researchers found the overall quality and safety of a neighborhood significantly contributed to the total minutes of their leisure time walking in a week [26]. However, little is known regarding the factors influencing the duration of their walking per occurrence. Generally, the duration depends on the distance and the speed of walking. It has been found the comfortable speed of walking in older adults is averaged to 1.3 m per second and associated with age, gender, and physical functions (e.g., dynamic balance and agility) [27, 28].

Neighborhood streets work as places not merely for passing through but also for communication and socialization. The streets make an important domain of age-friendly communities, providing places for older adults, especially those with restricted mobility, to regularly visit, get informed, commune with neighbors, meet friends and make new ones [29]. From this perspective, neighborhood streets have a strong social attribute, which needs to be promoted for active living. Specifically, the places of gathering along a street provide settings for socialization. These places typically offer seating furniture, which is also very welcoming to those engaging in physical activity. People meet there and may encourage each other to participate in active travel, physically and socially. Neighborhood social environments have been found playing a prominent role in shaping residents’ mental health but little is known regarding the social influence on their active travel [30, 31]. Moreover, most of the aforementioned studies were conducted in modern cities in western countries. Neighborhood streets and older adults’ active travel in eastern countries, especially those in ancient towns, need investigation.

### Ancient towns and active travel

The largest groups of older adults live in eastern countries, where a lot of ancient towns are well preserved and occupied [32]. Ancient towns can be defined as historically established settlements distinguished by their cultural heritage, traditional architecture and planning, reflecting the social, economic, and environmental practices of their time. Neighborhood streets in an ancient town typically have constrictions on vehicular traffic and thus provide good opportunities for older adults to safely use the streets as places of active travel.

The percentage of older adults in ancient towns has increased faster than in cities [33]. A recent trend is revitalizing ancient towns into characteristic towns for tourism-based retirement services [34]. It is reasonable to expect seeing more older adults in ancient towns. As an important domain of their daily-living environments, neighborhood streets need investigation to promote active living. However, most research on ancient towns, including those involving neighborhood streets, focuses on historic preservation, whereas research on active living are generally conducted in modern cities. The link between ancient towns and active living has been ignored.

To address the gap in research, we took Daokou ancient town in China as an example to investigate older adults’ active travel on neighborhood streets. Seeking to extend previous research on neighborhood streets as places of older adults’ active travel and social interaction [9], this paper focused on the travel duration. The aim is to identify environmental attributes promoting active travel. The questions are: (1) do the durations of older adults’ active travel vary by street? (2) If so, what are the characteristics of a street where they spend more time walking? It is hypothesized that older adults spend more time walking on the streets with more age- and social-friendly features.

## Methods

Empirical data on older pedestrians’ active travel and neighborhood streets were collected through on-site non-participant observation. It has been defined as a method of data collection via on-site observation. During the observation, researcher observes the street environments, older pedestrians, and their active travel in its natural setting without actively engaging or participating in the activities being studied. Statistical data analyses were conducted to investigate the differences in travel duration.

### Research site

**Fig. 1:**
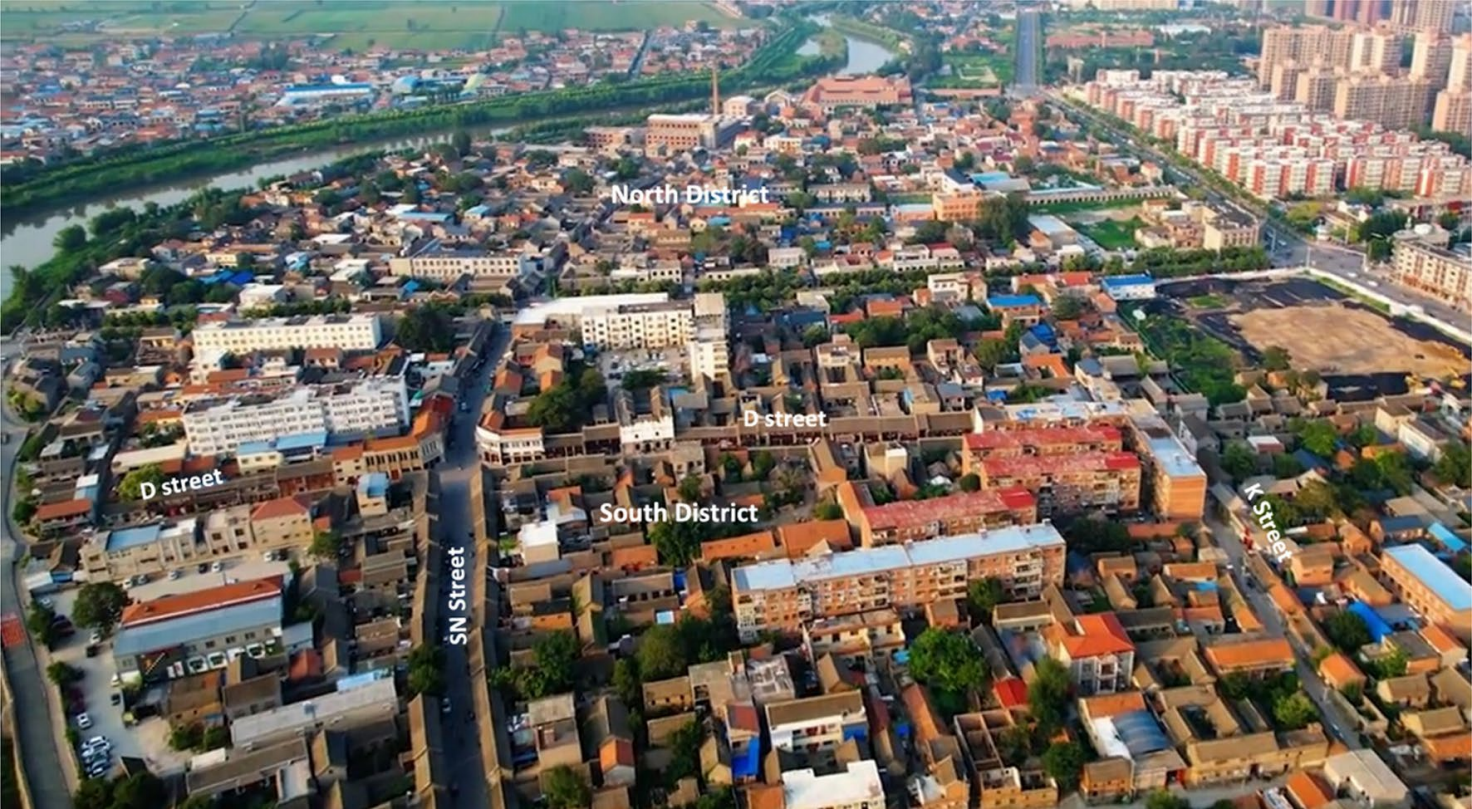
Aerial photo of the south district of Daokou Ancient Town

Daokou Ancient Town is one of the Nation Cultural Heritage Sites designated bythe National Cultural Heritage Administration in China. It has two districts (north and south districts) separated by a county-level avenue (Fig. 1). Due to restrictions on car traffic, no public transit is provided in the ancient town. People walk, cycle, or drive scooters (electric or motorized scooters for adults) for local transportation.

**Fig. 2:**
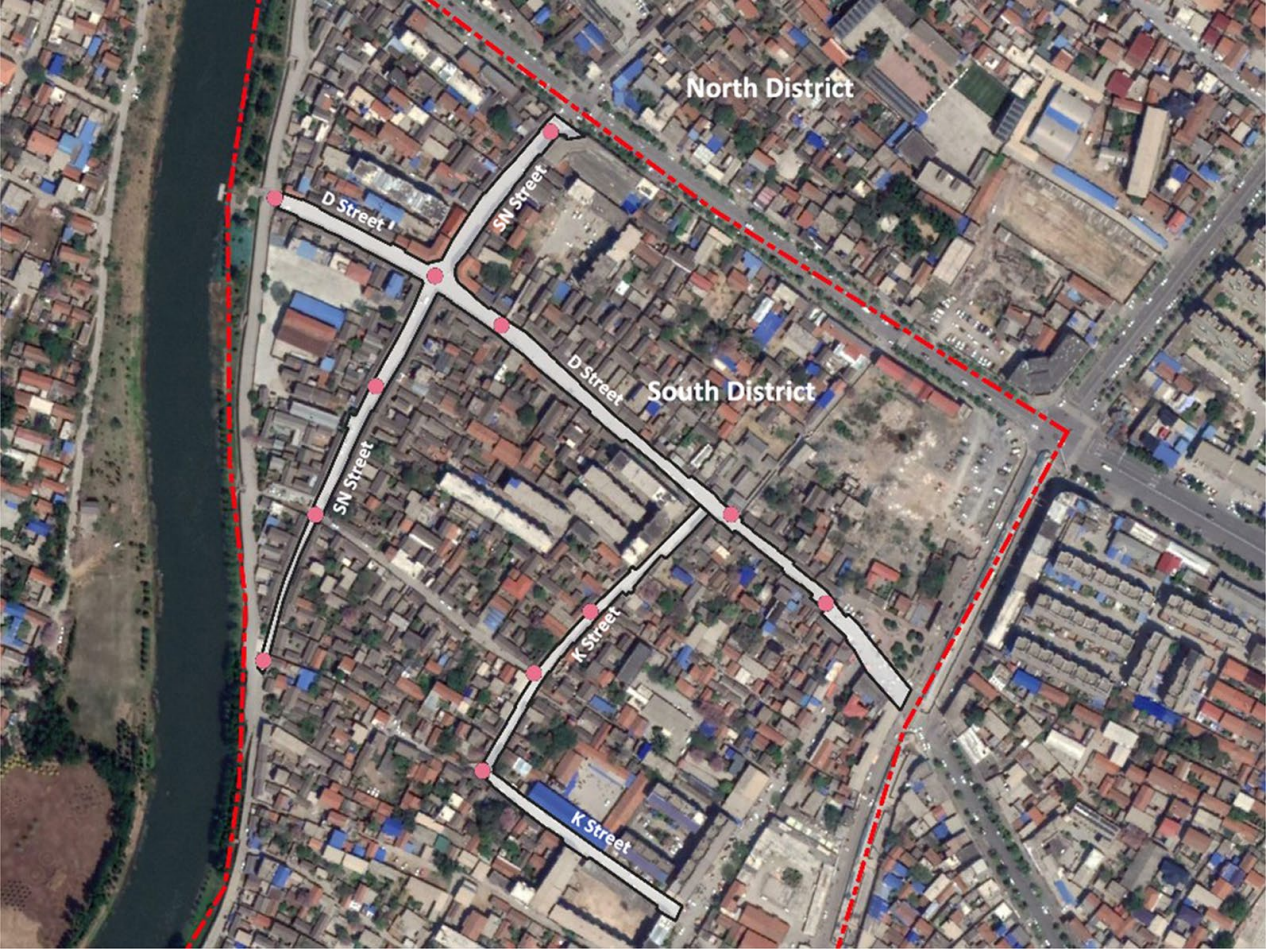
Streets in the south district of Daokou Ancient Town

**Fig. 3:**
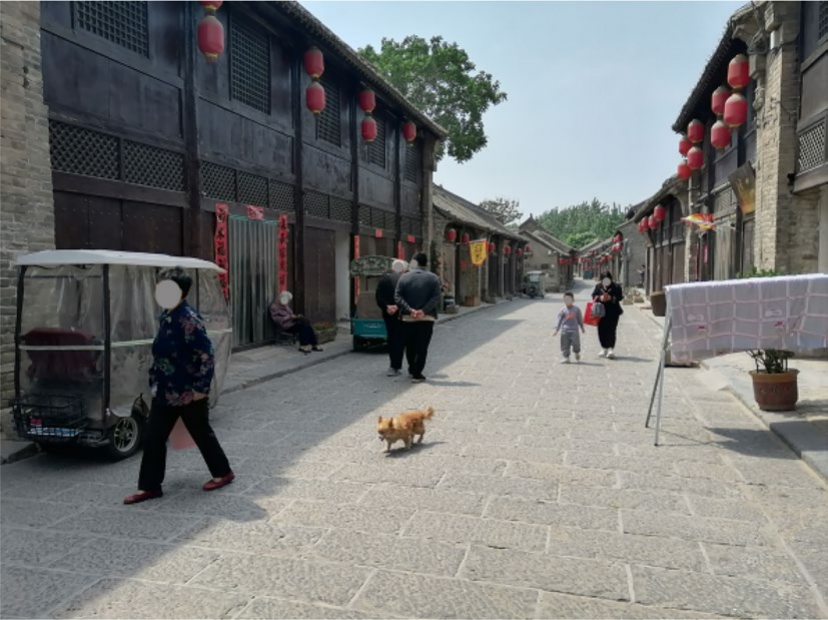
SN Street view

**Fig. 4:**
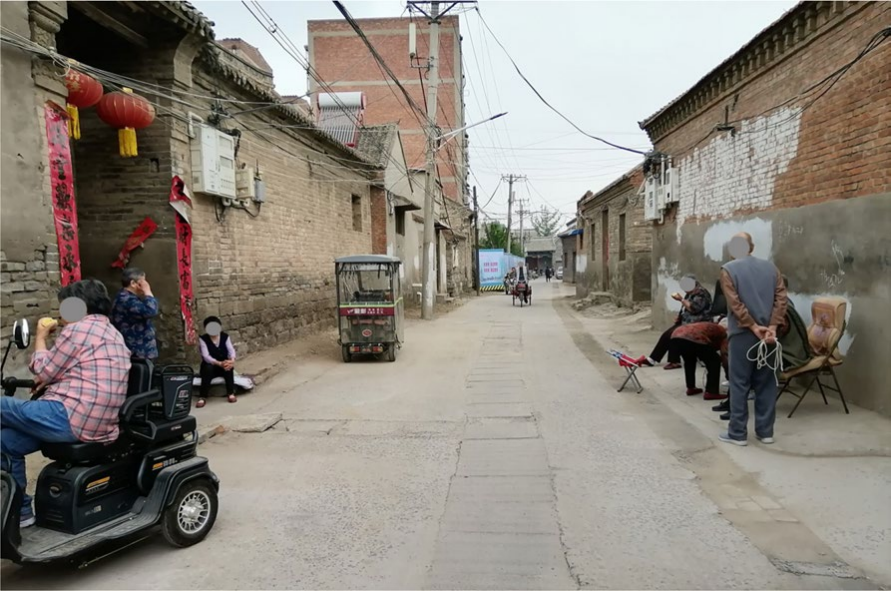
K Street view

**Fig. 5:**
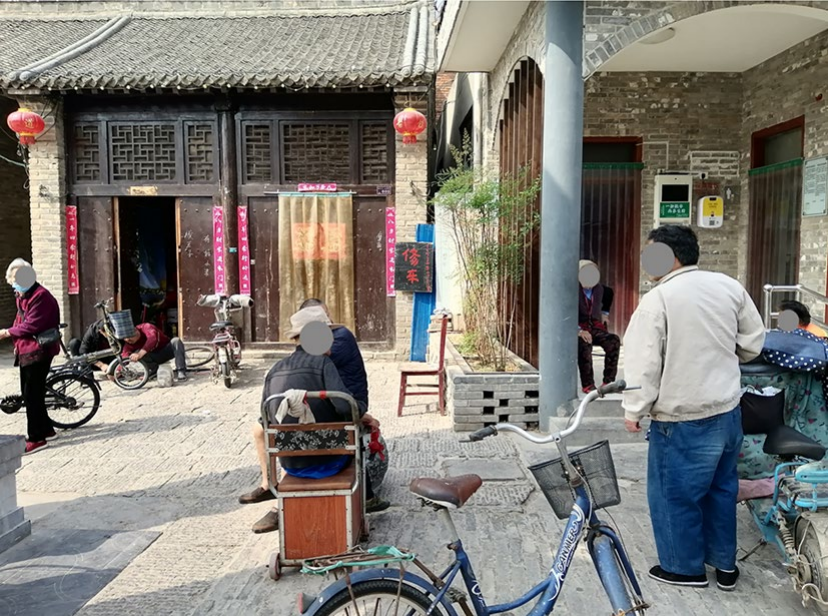
Place of gathering on the D Street

**Fig. 6:**
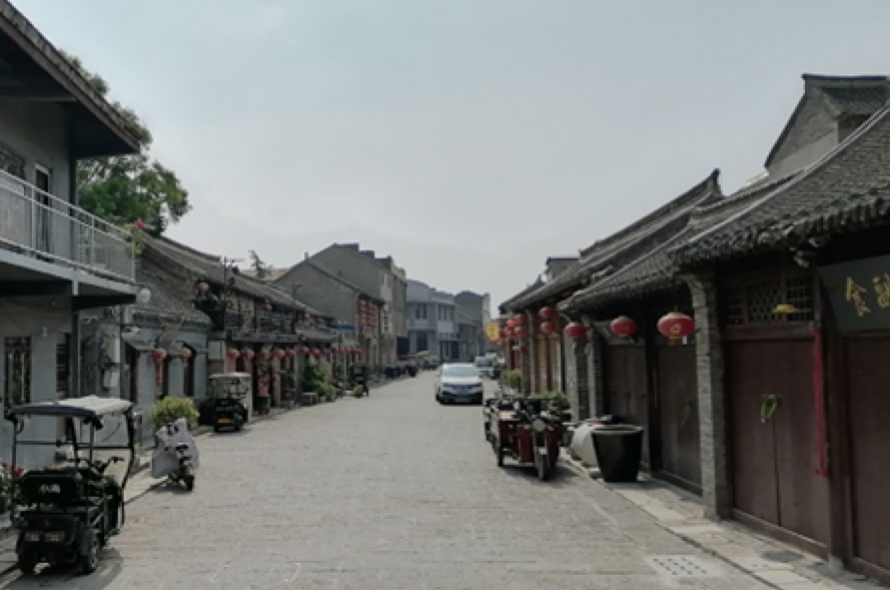
D Street view

Inside the south district, three neighborhood streets are connected with each other and have similar lengths (447, 396, and 387 m for the D, SN, and K Street, respectively) (Figs. 1, 2). With an average width of 6 m, these streets were originally built in Qing dynasty to provide services to visitors and connect local residential alleys. At that time, these streets shaped the major part of the local business system. In recent decades, the D and the SN Street have been used for both commercial and residential purposes, whereas the K Street has transformed into a residential street (Figs. 3, 4, 5, 6). Nine residential alleys (with an average width of 2.6 m) are directly connected to these streets. A total of 47 historic sites can be found along these streets, such as ancient temples and courtyards.

The D and the SN Street were renovated in 2019 to follow the recent trend of revitalizing ancient towns into characteristic towns for tourism. Stone-brick surface pavement, new drainage systems, and the rebuilding of historic forms on frontier building facades were applied. On the K Street, the most recent renovations were done in 2016, including concrete surface pavement and drainage system updates. Due to the COVID-19 pandemic, these streets weren’t open to tourists at the time of our data collection and provided a good opportunity to investigate local older adults’ active travel on neighborhood streets.

### Variables

Investigating the environmental context and the social attributes of active travel in older adults, this research included variables in multiple disciplines, such as environment, behavior, and gerontology. To better understand and organize these variables, the Social Ecological Model (SEM) was applied. This widely accepted model describes a structure of relationships among personal, social, environmental, and behavioral factors [35, 36]. Based on this model, this research grouped the independent variables into three categories: personal, social, and environmental factors.

Focusing on older pedestrians, active travel was accessed by location (street), mode (independent walking or walking with support such as a cane or walker), and duration (the minutes of a pedestrian’s presence on the street during observation). Considering the influence of mobility on travel duration, our data analysis focused on the pedestrians who walked independently without support. Due to limited data availability, the personal factors in research were perceived age (60 years or older), gender, and health, defined as the age, gender, and health status that a person was visually estimated to be on the basis of physical appearance [37].

Two social factors were included in research: social engagement during active travel and the time of active travel. Social engagement was investigated by whether engaging in social interaction (e.g., chatting with people, watching others playing chess or cards, group walking or other group activities) during the travel. Given the importance of the schedule for eating in older adults’ daily living, the time of active travel in research included four dayparts: early-morning (before breakfast), mid-morning (after breakfast), afternoon (after lunch), and evening (after dinner) (Table 1).

**Table 1:**
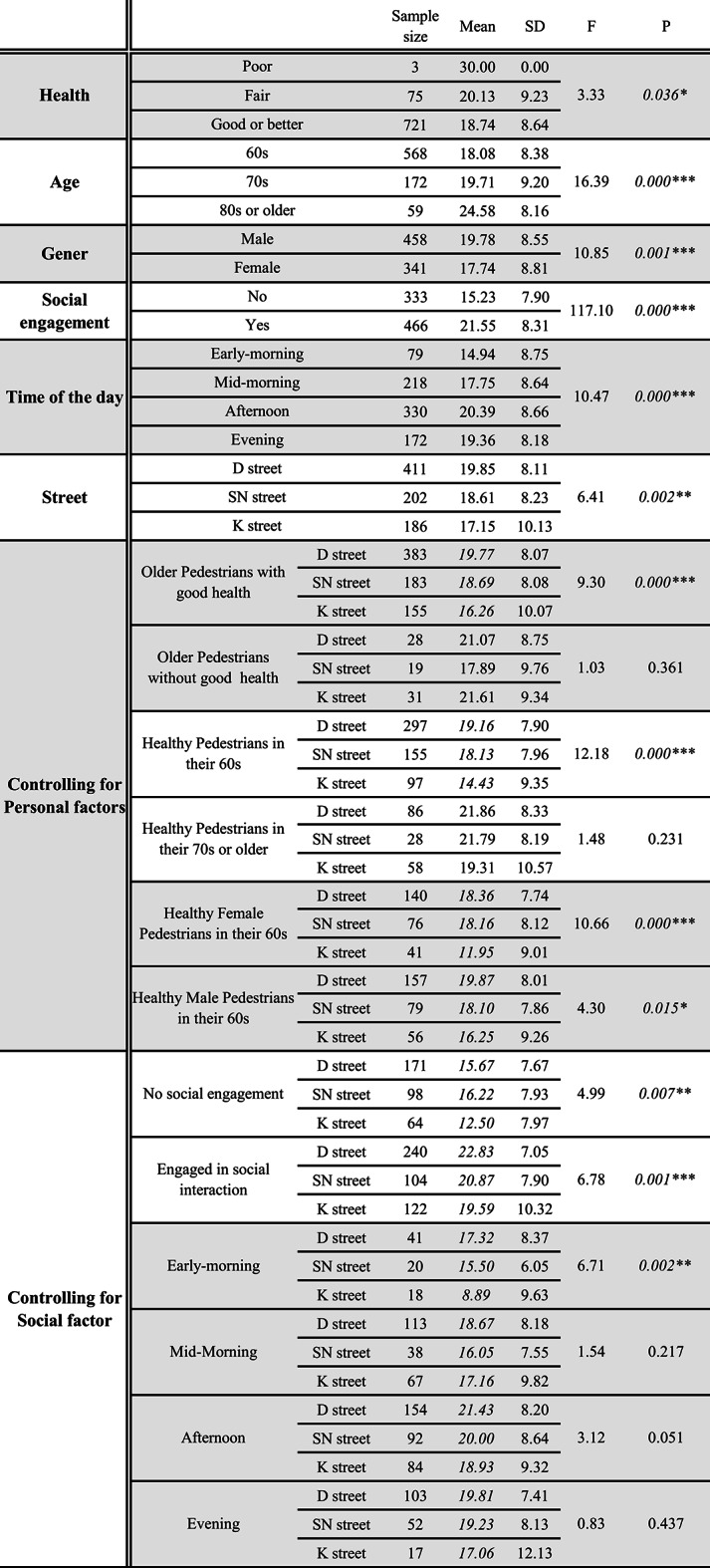
Older adults’ active travel duration by one-way ANOVA

Regarding the street environments, based on previous research on senior-friendly environments for walking, 20 features in four categories in terms of active-travel promotion were included: typology, motivators, functionality, and safety (Table 2) [16, 38]. Street type by function (commercial, residential, or mixed use), length, and width were included as the factors of street typology. Social-friendly features including the places of gathering and people of the same age group in active travel were included as motivators encouraging active travel. Environmental amenities were also considered as motivators encouraging people to join active travel, such as good landscaping and historic sites of interest to pedestrians. To measure the street’s physical functionality accommodating active travel, the variables of surface evenness, maintenance, seating furniture, restrooms, and signage were included in research. Regarding street safety, the variables of traffic volume, street lighting, security cameras, and safety perception were included.

**Table 2:**
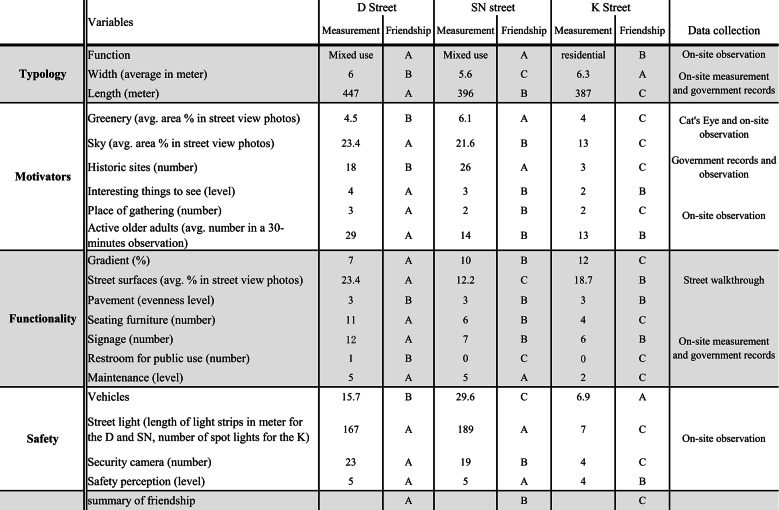
Neighborhood Street Affordance to Older Adults’ Active Travel

### Data collection

Two-level on-site observation was conducted by two researchers in April 2021. The observation collected data on the environment, people, and their behavior. The first level was conducted in the first week of the April and regarding street environmental factors. The second level was conducted in the following two weeks and focused on older pedestrians and their active travel. For data collection, we introduced the research aim, methods, and schedule to the local government via consent letters and phone calls and collected approval. This research was also reviewed and approved by a university-based institutional review board in China.

To collect data on street environments, we used a street investigation tool titled Cat’s Eye. It was supported by geographic positioning systems and computational vision technology and has been widely used in street design and planning projects in China. Data collected by Cat’s Eye has been demonstrated to be reliable [39]. By using this tool, we took digital photos of street view at eye level for every 20–25 m on these streets. A total of 54 street-view photos (18 of the D, 17 of the K, and 19 of the SN) were investigated, including the percentages of greenery, sky, buildings, and street surfaces on the photos. Laser meters (ISO9001 certified) were applied to measure the street environments. Street maintenance and surface evenness were rated by using a 5-point Likert Scale from low to high.

Given the general differences in daily living between weekdays and weekends and the possible influence of weather on active travel, the second-level observations were conducted for six sunny weekdays (two on each street). To represent a typical day, we used seven 30-minute observations: one for the early morning (6:30-7am), two for the mid-morning (9–9:30am and 10–10:30am), three for the afternoon (3–3:30pm, 4–4:30pm, and 5–5:30pm), and one for the evening (7–7:30pm). Three to five spots of observation were selected on each street to ensure the visibility of people and their behaviors (Fig. 2). Focusing on pedestrians with a perceived age of 60 years or older, researchers observed their active travel on the streets without participation or interruption. The information of one’s active travel duration on the street, social engagement, perceived age, gender, and health was recorded. To represent vehicular traffic, the number and types of vehicles (e.g., scooters, motorcycles, and cars) passing through the streets during observation were counted. Both researchers held graduate degrees in environmental design and received training in data collection at a local university. Coding was used by them consistently during the observation to ensure researchers’ similar decisions about similar events on different occasions. All pedestrians on the street were observed and the active travel of those with perceived age older than 60 years were recorded, including those who walk independently or with a cane or walker. The inter-observer agreement was almost perfect (Kappa > 0.8).

### Data analysis

Of each street feature, the friendship to older adults’ active travel was individually analyzed and ranked at three levels from A to C, with A the highest. Of the 20 features considered appliable to the friendship, rankings were summarized by street (Table 2). The average durationsof older pedestrians’ active travel on these streets (in a 30-minute observation) were categorized and compared by street, age, gender, health, social engagement, and daypart.

To clarify the differences in active travel duration by street, with attention to the personal and social factors, one-way ANOVA tests were conducted using the Statistical Package for Social Science (version 22.0). Based on the SEM, personal and social variables were analyzed in the first-level ANOVA tests, to identify those associated with significant differences in travel duration (p < = 0.05, two-tailed). To control for the personal or social variable(s) identified as being significant, the data was divided into sub-sets by the category of a significant variable. Using each of the subsets, second-level ANOVA tests were then applied to compare the travel durations by street. The streets where older pedestrians spent more time traveling were identified and discussed.

## Results

### Active travel and older pedestrians

Focusing on older pedestrians, a total of 871 cases of active travel were recorded during the observations. Of the 871, 799 walked independently and the rest walked with canes (51) or walkers (21). Of the 799, the perceived age was in the mid-60s on average and the majority (90%) looked healthy. The average duration of older adults’ active travel was 18.9 min in a 30-minute observation. Considering the lengths of these streets (447, 396, and 387 m) and the average walking speeds of older adults—which vary by age, gender, and health conditions, typically ranging from 0.8 to 1.3 m per second—older adults would need approximately 5 to 9 min to walk through these streets [40, 41]. However, their active travel durations were two to three times longer. They spent time doing more than just walking through the streets. During the process of active travel, they frequently stopped walking to watch or chat with others. Our data analyses found those who engaged in social activities had significantly longer travel durations than their peers (*p* < 0.01). Regarding the time of active travel, in the afternoons and evenings, they spent more time walking on the streets.

The number of older adults walked on the D Street was more than doubled than on the SN and K Street (an average of 29 vs. 14 and 13 in a 30-minute observation, respectively). Active travel durations on the D were the longest (ranging from 15.67 to 22.83 min in a 30-minute observation), whereas those on the K Street were the shortest (ranging from 8.89 to 21.61 min) (p < = 0.01) (Table 1). Although the street lengths slightly varied, the differences in travel duration (37% on average) were near to tripled than those in street length (13% on average).

Pedestrians aged 80 + had longer travel durations on the neighborhood streets than those in their 70s and 60s. So did the pedestrians apparently without good health (rated as poor or fair). According to our observation, they spent most of the time on the street watching or chatting instead of walking. During the observations, the distances they had walked were shorter than their peers and the physical intensity of their active travel was at a low level. Interestingly, more older men (458 out of 799) were observed on the streets than the women, and they also spent significantly more time walking on the streets (*p* < 0.01).

After controlling for significant personal or social variables, in the sub-groups of healthy older pedestrians in their 60s, for both females and males, irrespective of their social arrangements, travel durations were still significantly longer on the D and the SN Street than on the K (p < = 0.01). However, the differences were no longer statistically significant in the subsets of people aged 70 years or older (70+) or without good health, and in the mid-morning and evening observations (*p* > 0.05).

### Neighborhood streets

Compared with the K Street, the environments on the D and the SN Street were more friendly to older pedestrians and their active travel including both physical and social activities (Table 2). Thirteen of the 20 environmental features of the D were ranked at the highest level of friendship, whereas six on the SN and two on the K were ranked at this level (Figs. 3, 4). Among the three streets, only the D provided restrooms for public use. Of the D, the gradient was lower and the views were more open to the sky. Importantly, pedestrians on the D had more opportunities to find seating furniture and places of gathering (Figs. 5, 6).

Among the three streets, the K had the widest overall width on average (6.3 m) but 11 features of it were ranked at the lowest level of friendship, including the highest gradient (12%), the lowest levels of maintenance, greenery, and interesting things to see. Differently, with the widths narrowed up to 3.7 m and averaging to 5.6 m, the SN Street had more historic sites of interest to pedestrians, more greenery, and the largest volume of vehicular traffic (including cycles, scooters, motorcycles, and a few cars) among the three streets.

## Discussion

### Supportive street environments

Findings of this research highlighted the importance of street environmental quality, instead of the size, in driving the decision on active travel duration. Among the three streets, K Street had the widest spaces for pedestrians. However, in the relatively spacious environments, older pedestrians’ active travel durations were the shortest. In contrast, width of the SN Street was narrowed up to 3.7 m but older pedestrians spent more time travelling on it. Compared with the K, the SN had better maintenance, more sky views, street lights, safety cameras, and historic sites of interest to pedestrians. This may be associated with its mixed use for both commercial and residential purposes [23]. Similarly, with the highest level of friendship, the D Street was used for multiple purposes and provided more seating furniture, signages, places of gathering, and interesting things to see. More older pedestrians were seen on the D and they had the longest active travel duration on average. Environmental details designed to support walking, such as pedestrian furniture and amenities, can increase active-travel duration, which, in turn, may drive further enhancements to the environment [24]. Future research is needed to disentangle the direction and strength of these relationships more definitively.

Vehicular traffic on the neighborhood streets can be viewed as a double-edged sword, in terms of promoting active travel in older adults. On K Street, on average, less than 7 vehicles (e.g., electric motorcycles or cars) were observed in a 30-minute observation. Although the environments were quieter and safer from vehicular traffic, older adults’ active travel on this street was significantly shorter in duration. One of the possible reasons could be the missing of interesting things to see since a few things happened on the street [42]. Differently, the volume of vehicular traffic on the SN Street was the largest (an average of 29.6 vehicles observed in a 30-minute observation). Although safety from vehicular traffic could be a concern to older pedestrians, they spent more time traveling on this street. However, the longest durations were on the D Street, where a medium volume of vehicular traffic (an average of 15.7 vehicles observed in a 30-minute observation) was observed. Together with other environmental factors, the appropriate volume of vehicular traffic on neighborhood streets should be investigated if promoting active travel in older adults.

### Social factors

Confirming previous research, our finding clarified the contribution of social engagement to active travel [43]. Based on our observation, about three-fifths of the older pedestrians engaged in social interactions and had longer durations of active travel. On the streets providing physical support to older adults, such as wide, well-maintained sidewalks and adequate lighting, they were more likely to engage in social interactions [9]. These findings support each other and highlight the interrelationship between the social and physical attributes of active travel, in the context of neighborhood street environments. To promote neighborhood streets as places of active living for older adults, street design or renovation should balance the physical accessibility of street al.ongside its social functionality. A street designed to meet the requirements of a walkway or standard cycles would be insufficient to meet the needs of social engagement. However, a resting point encouraging movement can also be built as a place of gathering for social engagement. For instance, the SN Street was renovated to be more attractive to tourists but no seating furniture was provided. To address the need for seating furniture, residents helped themselves with used chairs located near their residence entrances. According to our observation, these rest areas had been used as places of gathering by older adults.

Regarding the daypart, older adultsengaged in longer active travel in the afternoons and evenings. Street influences on their active travel were significant in the early mornings and afternoons. In the context of local cultures, they may have more time to travel during the generally ‘longer’ hours in the afternoons and thus more likely to be influenced by the environments. As part of the traditional Eastern cultures, many older adults live with their adult children and grandchildren in China. They may shop for breakfast or help send kids to school in the mornings. Of them, schedules may be tight in the mornings, and thus spent less time to walk on the streets. Moreover, the early-morning shopping typically happened on neighborhood commercial streets (the D and SN Street) so they were more likely to walk there. In the evenings, adult family members are back at home and older adults may have more free time to walk outside. These help to better understand the differences in active travel duration in terms of daypart.

Our findings demonstrate that social engagement positively influences active travel, aligning with previous research conducted in Western contexts. However, the observed impact of daypart on active travel may reflect characteristics of Eastern lifestyles and warrants further investigation in the context of Western cultures.

### Active travel in ‘advanced’ older adults and the males

Most older adults seen on the neighborhood streets were in their 60–70 s. It was rare to see those aged 80+ (7%), cane or walker users (8%) on the streets. However, the 80 + population has been increasing the fastest among the older populations and is expected to reach 19% in 2050 [44]. At that time, older adults on neighborhood streets are more likely to need a cane or walker for active travel. Future street designs and developments should be more welcoming to them.

Among the group of pedestrians aged 70 + and those without good health, the differences in active travel duration weren’t significant by street. Compared with the environments, age-related mobility and health declines may have a stronger impact on older adults and their active travel. Although these 70 + were rare to be seen on neighborhood streets, they spent significantly more time on the streets. They may not engage in the suggested moderately-intensive physical activity such as brisk walking but the time spent on neighborhood streets offers opportunities for them to access nature and meet people. Benefiting health, these are their contacts with the world outside home and should be promoted.

For disabled older adults, the field of active travel might be perceived as problematic since they may be cast as immobile by the constraints that an ableist society places on them. As examples of/synonyms for active travel, walking and cycling may be overwhelmingly cited. The modes qualifying as active travel should include more, such as travel by manual wheelchair and cycling using power-assisted cycles [5]. To older adults, manual or power-assisted wheelchair running and cycling are both a mobility aid and a form of transport. Active travel in older adults needs to reach its potential of inclusion. It was found that tricycling was popular in older adults and safer than bicycling [9]. Today, more cycles are power-assisted with electricity, whereas a rise in cycling accidents has been found in older e-bike users [45]. The advantages and disadvantages of electric vehicles should be balanced for active travel.

Moreover, compared with older women, older men spent more time actively traveling around home on neighborhood streets. It is interesting since men in their working age generally spend less time at home than women. Things may change after their retirement. More environmental investigation is suggested if promoting gender adjustments on neighborhood streets for active travel.

### Implications for practice

Findings from this research suggest a clear need for active travel promotion targeting upstream neighborhood contexts, including the improvement of street conditions to foster social integration among older residents. In ancient towns, the widely applied restrictions on vehicular traffic help to facilitate active travel. Street environmental quality, places of gathering, amenities, and traffic density are important attributes determining older adults’ active travel duration. To help them reach the suggested level of active living, both the social and physical attributes of active travel should be promoted in street, transport, and public health interventions.

New and emerging technologies may challenge neighborhood renovation and intervention. For the development of an ancient town and its historic preservation, more attention towards local networks and lighter infrastructure is needed in street design and planning, health promotion, and transport engineering. Looking beyond ancient towns, a new dialogue could develop between environment and health. Having noticed the integration of physical and social activities in older adults’ active travel, we may consider how this integration might fit into an enriched health and environmental paradigm.

### Limitations and next steps

It was a cross-sectional study undertaken in China and had no causal conclusions. Personal and social variables such as one’s biological age, medical records, and living arrangements may affect the findings. The same pedestrian may be observed more than once during the on-site observation for six weekdays. Special attention should be paid to older adults with advanced ages, health issues, and restricted mobility. Active travel by manual wheelchair or power-assisted cycles needs to be investigated.

Next, experimental or longitudinal methods with culturally balanced samples should be considered to expand our understanding of healthy and active aging. Intercept studies could gather real-time feedback from older adults, helping to identify specific environmental features influencing their active travel. Qualitative research, like interviews or focus groups, would provide deeper insights into how street design affects safety, comfort, and social interaction among older adults. Additionally, co-production and co-design initiatives involving older adults would ensure neighborhood streets better meet their needs.

## Conclusions

Age- and social-friendly neighborhood streets contributed to longer durations of active travel in older adults. Social engagement plays an important role in older adults’ active travel. To help them achieve the recommended level of active living, street affordances for both the social and physical attributes of active travel should be created by policymakers, planners, and street designers. Street interventions applied in ancient towns should go beyond historic preservation and pay more attention to the need for active living.

## Data Availability

The datasets used and analysed during the current study available from the corresponding author on reasonable request.

## Abbreviations

ANOVA: Analysis of variance
ISO: International Organization for Standardization
SEM: Social ecological model
